# Severe intraoperative bleeding predicts the risk of perioperative blood transfusion after robot-assisted radical prostatectomy

**DOI:** 10.1007/s11701-021-01262-z

**Published:** 2021-06-15

**Authors:** Antonio Benito Porcaro, Riccardo Rizzetto, Nelia Amigoni, Alessandro Tafuri, Aliasger Shakir, Leone Tiso, Clara Cerrato, Stefano Zecchini Antoniolli, Vincenzo Lacola, Alessandra Gozzo, Katia Odorizzi, Rossella Orlando, Giacomo Di Filippo, Matteo Brunelli, Filippo Migliorini, Vincenzo De Marco, Walter Artibani, Maria Angela Cerruto, Alessandro Antonelli

**Affiliations:** 1 Department of Urology, Azienda Ospedaliera Universitaria Integrata, University of Verona, Piazzale Stefani, 137126 Verona, Italy; 2 Department of Neuroscience, Imaging and Clinical Sciences, G. D’Annunzio University, Chieti, Italy; 3 USC Institute of Urology and Catherine and Joseph Aresty Department of Urology, Keck School of Medicine, University of Southern California (USC), Los Angeles, CA USA; 4 Department of General and Hepatobiliary Surgery, Azienda Ospedaliera Universitaria Integrata, University of Verona, Verona, Italy; 5 Department of Pathology, Azienda Ospedaliera Universitaria Integrata, University of Verona, Verona, Italy

**Keywords:** Prostate cancer, Robot-assisted radical prostatectomy, Blood transfusion, Complications, Clavien–Dindo grading system of complications

## Abstract

To evaluate potential factors associated with the risk of perioperative blood transfusion (PBT) with implications on length of hospital stay (LOHS) and major post-operative complications in patients who underwent robot-assisted radical prostatectomy (RARP) as a primary treatment for prostate cancer (PCa). In a period ranging from January 2013 to August 2019, 980 consecutive patients who underwent RARP were retrospectively evaluated. Clinical factors such as intraoperative blood loss were evaluated. The association of factors with the risk of PBT was investigated by statistical methods. Overall, PBT was necessary in 39 patients (4%) in whom four were intraoperatively. Positive surgical margins, operating time and intraoperative blood loss were associated with perioperative blood transfusion on univariate analysis. On multivariate analysis, the risk of PBT was predicted by intraoperative blood loss (odds ratio, OR 1.002; 95% CI 1.001–1.002; *p* < 0.0001), which was associated with prolonged operating time and elevated body mass index (BMI). PBT was associated with delayed LOHS and Clavien–Dindo complications > 2. In patients undergoing RARP as a primary treatment for PCa, the risk of PBT represented a rare event that was predicted by severe intraoperative bleeding, which was associated with increased BMI as well as with prolonged operating time. In patients who received a PBT, prolonged LOHS as well as an elevated risk of major Clavien–Dindo complications were seen.

**Supplementary Information:**

The online version contains supplementary material available at 10.1007/s11701-021-01262-z.

## Introduction

Prostate cancer (PCa) is one of the most frequent tumors affecting aging males as well as challenging to treat. Its treatment may involve multiple specialists that include urologists, radiotherapists and oncologists [1, 2]. When life expectancy is above 10 years, guidelines recommend several treatment modalities for non-metastatic PCa, which include active surveillance, surgery and radiation therapy; furthermore, multi-modality therapy is recommended when the disease is locally advanced [1, 2].

In urologic tertiary referral centers, robot-assisted radical prostatectomy (RARP) is the preferred surgical approach for prostate cancer [1, 2]. Robotic surgery has been shown to accelerate stress recovery post-operatively [3], and as with any laparoscopic approach, due to the insufflation of CO_2_, overcomes intraoperative severe bleeding which is one of the most frequent major intraoperative complications of the open approach [4].

The aim of this study was to evaluate factors associated with the risk of perioperative blood transfusion with relative implications on the overall post-operative course in patients undergoing RARP.

## Materials and methods

### Study features

The study was retrospective and approved by Institutional Review Board. Data were prospectively collected for each patient who provided informed-signed consent. RARP was delivered by the da Vinci Robot System (Intuitive Surgical, Inc., Sunnyvale, CA, USA) through the trans-peritoneal approach with antegrade prostatic dissection [5]. Operations were performed by surgeons who were classified into high and low volumes (> 100) according to a study reporting an initial reduction in complications and blood loss rate after 100 cases were performed [6]. In the low- and intermediate-risk categories, ePLND was performed according to EAU recommendations and factors predicting tumor upgrading [7–9]. As previously reported, lymph node dissection was developed according to a standard template that included lymph nodes involving the external iliac, obturator, Cloquet’s and Marcille’s anatomical regions [10, 11]. Prophylaxis of deep venous thrombosis with low molecular weight heparin was utilized until post-operative day 28 in all patients who underwent ePLND or had comorbidity risk factors. According to an internal protocol, patients who had an uneventful post-operative course were discharged on post-op day 4 with the catheter, which was then removed on the 12th post-operative day in an outpatient setting, without radiological controls. Patients under androgen deprivation or with previous treatments for PCa were excluded.

The clinical factors that were evaluated included plasma levels of prostate-specific antigen (PSA; ng/mL), age (years), body mass index (BMI; kg/m^2^), total prostate volume (PV; mL), rate of biopsy positive cores (BPC; percentage). Clinically, tumors were graded and staged according to the International Society of Urologic Pathology (ISUP) and TNM system, respectively; furthermore, patients were categorized into risk classes according to recommendations [1, 2]. Specimens were evaluated for tumor grade and stage, surgical margins status, number of removed and metastatic nodes by our dedicated pathologist [1, 2, 12, 13]. Perioperative surgical risk was evaluated by the American Society of Anesthesiologists (ASA) score system [14]. Operating time (OT), which was measured in minutes, was calculated as the interval between incision and suture of the skin. Intraoperative blood loss (BL) was collected during surgery through the suction canister and specified after surgery in each surgical report evaluated and measured in milliliters (mL). Postoperative surgical complications were graded according to the Clavien–Dindo score system respecting Martin’s criteria, as suggested by EAU guidelines [1, 4, 13, 15]. Clavien–Dindo complications were also evaluated as major (greater than 2) as well as minor (up to grade 2). All patients were followed for complications and hospital readmission after discharge for a period of 6 months.

### Statistical methods

The aim of this study is to investigate the association of several factors with the risk of blood transfusion, which included both intra- or post-operative blood transfusion events. Additionally, we investigated whether transfusions were related to other post-operative complications or length of stay. Descriptive statistics of the patient population was computed. Distributions of continuous variables were evaluated by means (standard deviation, SD) and medians (interquartile range, IQR), as well. Categorial variables were assessed as frequencies (percentages). Associations of clinical, pathological, and perioperative factors with perioperative blood transfusion were assessed by correlation analysis. The association of significant covariates with the risk of perioperative blood transfusion was evaluated by the logistic regression model (univariate and multivariate analysis). Associations among continuous covariate(s) predicting the risk of perioperative blood transfusion were evaluated by correlation analysis and then assessed by the linear regression model (univariate and multivariate analysis). The effect of blood transfusion on LOHS and major post-operative complications (Clavien–Dindo score > 2) were evaluated by the logistic regression model (univariate and multivariate analysis). The software used to run the analysis was IBM-SPSS version 26. All tests were two-sided with *p* < 0.05 considered to indicate statistical significance.

## Results

### Risk of blood transfusion

In a period ranging from January 2013 to August 2019, 980 consecutive patients underwent RARP. According to the D’Amico classification, 298 patients were low risk (30.4%), 520 intermediate risk (53.1%) and 162 high risk (16.5%). 96 cases were ASA score 1 (9.8%), 805 ASA score 2 (82.1%) and 79 ASA score 3–4 (8.1%). Extended pelvic lymph node dissection (ePLND) was performed in 581 patients (59.3%). The median (IQR) number of removed lymph nodes was 26 (21–32). Overall, perioperative blood transfusion was necessary in 39 patients (4%); 4 intraoperatively, 35 post-operatively. The median number of RBCU transfused was 2.4. Clavien–Dindo complications were classified as grade 1 in 142 cases (14,5%), grade 2 in 65 (6.6%), grade 3a in 16 (1.6), grade 3b in 15 (1.5%) and 4a in 2 (0.2%). Table 1 shows the demographics of the studied cohort.

**Table 1:** Demographics of the patient population who underwent robot-assisted radical prostatectomy (RARP; *n* = 980)

Clinical features	Mean (SD) or number (%)	Median (IQR)
Age (years)	64.5 (6.6)	65 (60–70)
Body mass index (BMI; kg/m^2^)	26 (3.1)	25.9 (23.8–28)
Prostate-specific antigen (PSA; ng/mL)	7.9 (7.3)	6.4 (4.9–8,8)
Prostate volume (PV; mL)	42.1 (17.6)	40 (30–50)
Biopsy positive cores (BPC; %)	34.4 (21.4)	29 (17.3–47)
Clinical stage (cT)		
cT1	658 (67.1)	
cT2	295 (30.1)	
cT3	27 (2.8)	
Clinical nodal stage (cN)		
cN0	944 896.3)	
cN1	36 (3.7)	
ISUP		
1	410 (41.8)	
2	308 (31.4)	
3	157 (16)	
4	87 (8.8)	
5	18 (1.8)	
Pathological features		
Prostate weight (PW; gr)	54.8 (18.8)	50 (41.3–64.8)
ISUP		
1	138 (14)	
2	377 (38.2)	
3	270 (27.3)	
4	136 (13.8)	
5	59 (6)	
Pathological stage (pT)		
pT2	772 (78.1)	
pT3a	96 (9.7)	
pT3b	112 (11.3)	
Pathological nodal stage (pN)		
pNx	399 (40.4)	
pN0	516 (52.2)	
pN1	65 (6.6)	
Positive surgical margins (PSM)		
No	725 (73.4)	
Yes	255 (25.8)	
Perioperative features		
Extended pelvic lymph node dissection (ePLND)		
No	399 (40.7)	
Yes	581 (59.3)	
Nerve sparing surgery (NSS)		
No	791 (80.7)	
Yes	189 (19.3)	
High-volume surgeon (HVS)		
No	422 (43.1)	
Yes	558 (56.9)	
Operating time (OT; minutes)	209.4 (53.6)	210 (170–245)
Intraoperative blood lost (BL; mL)	372.8 (321.4)	300 (200–470)
Discharge day (DD; days)	5 (1.9)	4 (4–5)
Readmission (RAD)		
No	951 (97)	
Yes	29 (3)	

*ISUP* International Society of Urologic Pathology prostate cancer (PCA) tumor-grade group system, *SD* standard deviation, *IQR* interquartile range

### Factors associated with the risk of blood transfusion

**Fig. 1:**
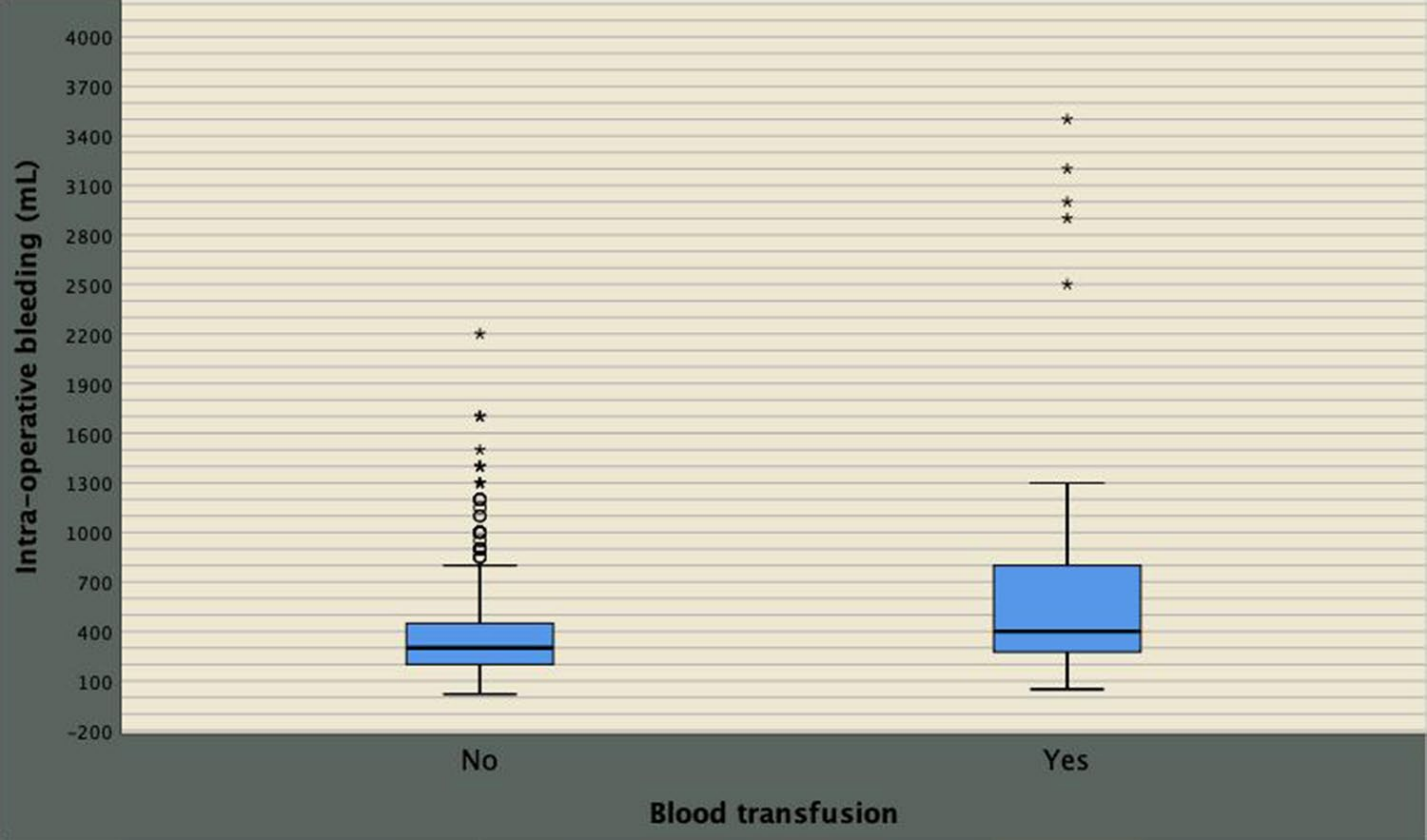
Scatterplot comparing intraoperative blood loss between not-transfused and transfused patients who showed more severe intraoperative bleeding (median 400 mL; interquartile range (IQR): 250–800 mL) compared with the not-transfused group (median 300 mL; IQR: 200–450 mL). On multivariate analysis, BL was the only independent factor predicting the risk of blood transfusion (odds ratio, OR 1.002; 95% CI 1.001–1.002; *p* < 0.0001). See Supplementary Table 1 for further details. When intraoperative blood loss was categorized by quartiles, the risk of blood transfusion was significantly predicted only for severe intraoperative bleeding, which occurred for values above the third quartile (OR 2.977; 95% CI 1.242–7.134; *p* = 0.014)

Positive surgical margins, prolonged operating time and intraoperative blood loss were associated with perioperative blood transfusion, as shown in Table 2. However, on multivariate analysis, only intraoperative blood loss predicted such risk (odds ratio, OR 1.002; 95% CI 1.001–1.002; *p* < 0.0001). As depicted in Fig. 1, intraoperative blood loss was significantly higher in transfused patients (median 400 mL; interquartile range, IQR 250–800 mL) compared with not-transfused cases (median 300 mL; IQR 200–450 mL). When intraoperative blood loss was categorized by quartiles, the risk of blood transfusion was significantly predicted only for severe intraoperative bleeding, which occurred for values above the third quartile (OR 2.977; 95% CI 1.242–7.134; *p* = 0.014).

**Table 2:** Analysis of factors associated with the risk of perioperative blood transfusion in patients undergoing robot-assisted radical prostatectomy (*n* = 980)

	Correlation analysis	Univariate analysis (*)	Multivariate analysis (*)
Statistics	*r* (*p *value)	OR (95% CI; *p *value)	OR (95%; *p *value)
Age	0.024 (0.448)		
BMI	− 0.009 (0.765)		
PSA	0.022 (0.490)		
PV	0.021 (0.512)		
BPC	0.024 (0.456)		
cT	0.040 (0.208)		
cN	− 0.012 (0.707)		
ISUP	0.030 (0.350)		
PW	0.000 (0.994)		
ISUP	0.009 (0.776)		
pT3	0.048 (0.137)		
pN	− 0.009 (0.770)		
PSM	0.070 (0.029)	2.043 (1.062–3.933; 0.032)	1.658 (0.822–3.344; 0.158)
ePLND	0.009 (0.770)		
NSS	− 0.033 (0.297)		
HVS	0.019 (0.554)		
OT	0.064 (0.044)	1.006 (1.000–1.012; 0.045)	1.000 (0.994–1.007; 0.876)
BL	0.256 (< 0.0001)	1.002 (1.001–1.002; < 0.0001)	1.002 (1.001–1.002; < 0.0001)

*r* Pearson’s correlation coefficient, *OR* odds ratio, *CI* confidence interval, (*) by logistic regression; see also Table 1

### Factors associated with intraoperative blood loss

**Fig. 2:**
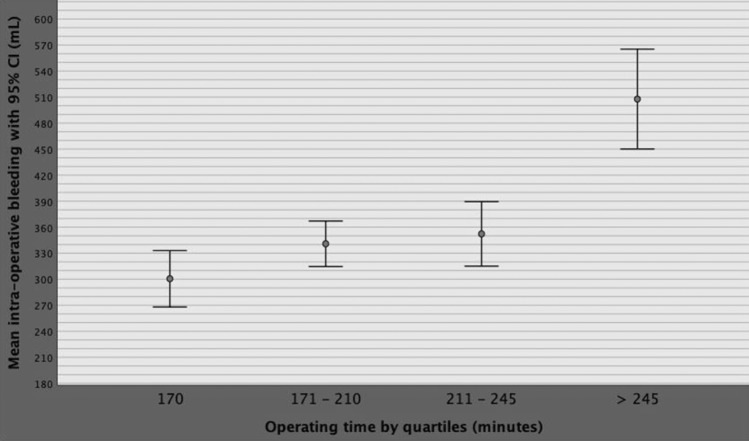
Mean blood loss distributions with 95% CI by operating time quartiles. As shown in the diagram and reported in Supplementary Table 2, severe intraoperative bleeding was significantly associated with operating time values above the third quartile, which was 245 min

**Fig. 3:**
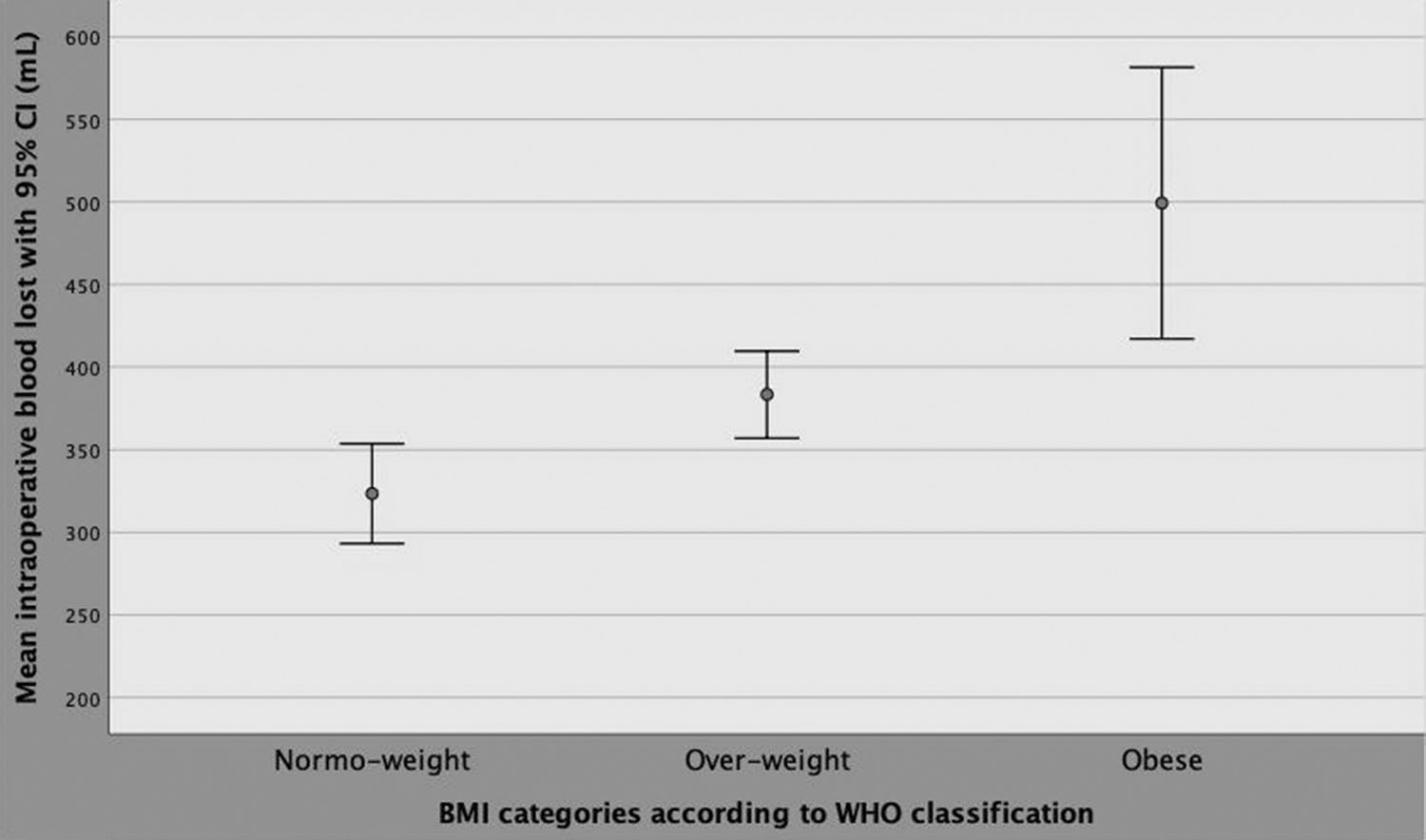
The diagram shows a significant linear positive association between intra-operative bleeding and BMI categories; as depicted, mean intraoperative blood loss increased through overweight up to obese patients compared with normo-weight subjects

**Fig. 4:**
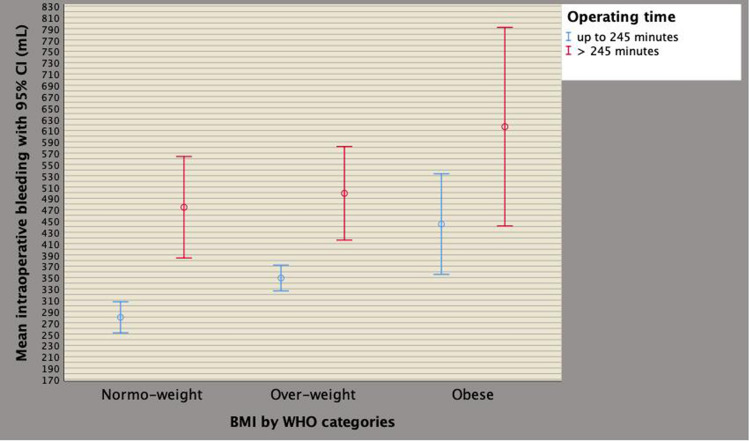
Independent associations of perioperative factors including operating time, blood loss and BMI. Although intraoperative bleeding increased along BMI categories, it severely worsened when operating times were prolonged beyond a limit of four hours. See Supplementary Table 2 and results section for further details

Although BMI, PSA, PV, PW, pT3, PSM, OT and high surgical volume per surgeon was correlated and associated with intraoperative blood loss on univariate analysis, only BMI and OT were independent predictors, as reported in Supplementary Table 1. BMI was then categorized according to WHO categories and OT by quartiles, as reported in Table 1 [16]. As detailed in Supplementary Table 2 and Fig. 2, mean intraoperative blood loss significantly increased for overweight and obese categories as well as for operating times above the third quartile (245 min), on multivariate analysis. Figure 3 depicts the linear positive association of blood loss along BMI categories. Blood loss was stratified according WHO BMI categorized including including normo-weight patients (BMI < 25 kg/m^2^), over-weight cases (BMI between 25 and 29.9 kg/m^2^) as well as obese subjects (BMI above 29.9 kg/m^2^). The diagram shows a significant linear positive association between intra-operative bleeding and BMI categories; as depicted, mean intraoperative blood loss increased through overweight up to obese patients compared with normo-weight subjects. The independent associations between operating time, blood loss and BMI are illustrated in Fig. 4, which shows that although intraoperative bleeding increased along BMI categories, it severely worsened when operating times were prolonged beyond the limit of four hours.

The distribution of complications in the 39 patients who received a PBT according to CDS was reported in the Supplementary Table 3. Clinical, perioperative, and pathological characteristics of these patients categorized according to CD < 3 and > 2 have been reported in Supplementary table 4.

### Post-operative course in patients undergoing blood transfusions

**Fig. 5:**
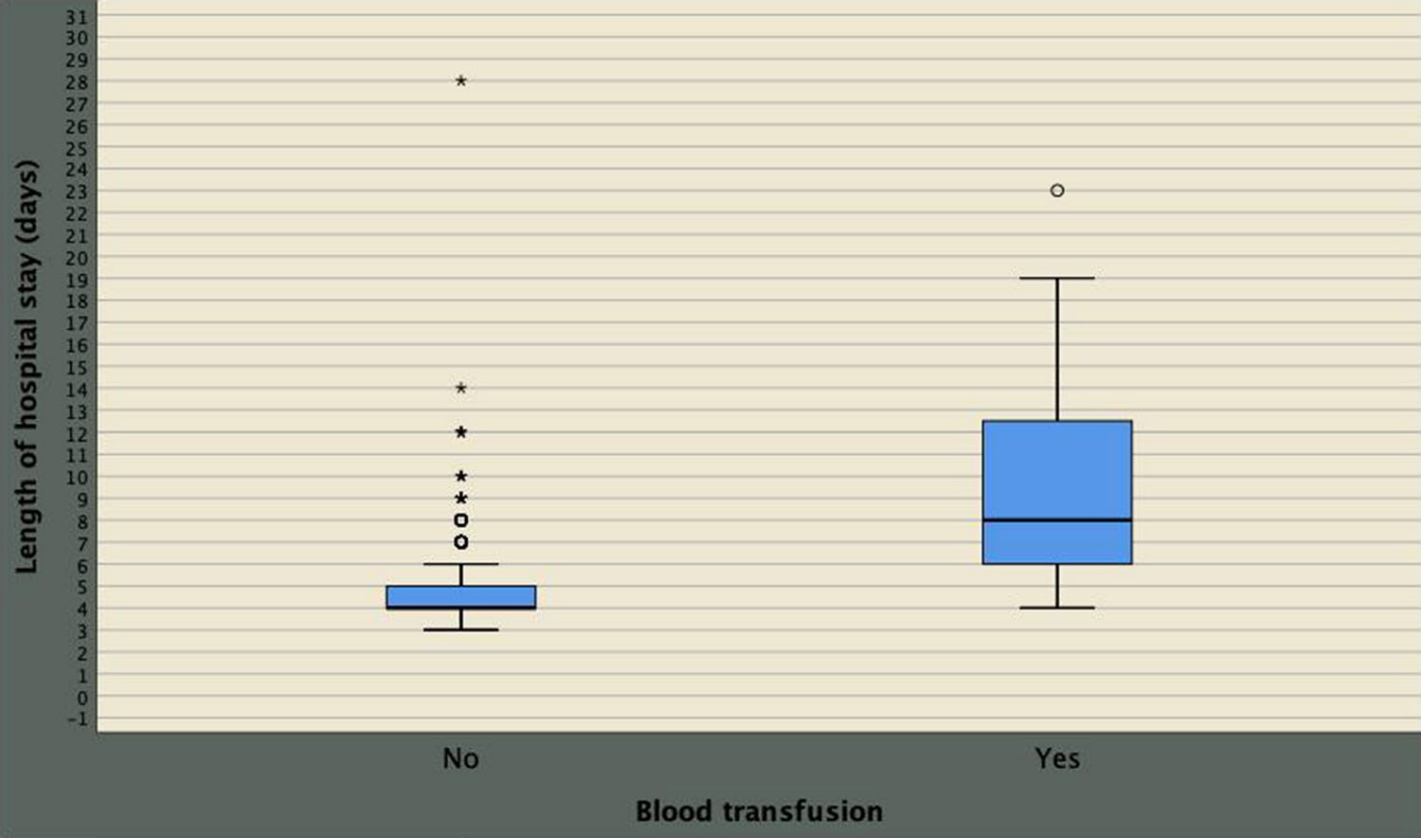
Blood transfusion is associated with the risk of prolonged length of hospital stay in 980 patients who underwent robot-assisted radical prostatectomy as a primary treatment for prostate cancer (corrected odds ratio, OR 1.633 with 95% CI ranging from 1.411 to 1.889)

**Fig. 6:**
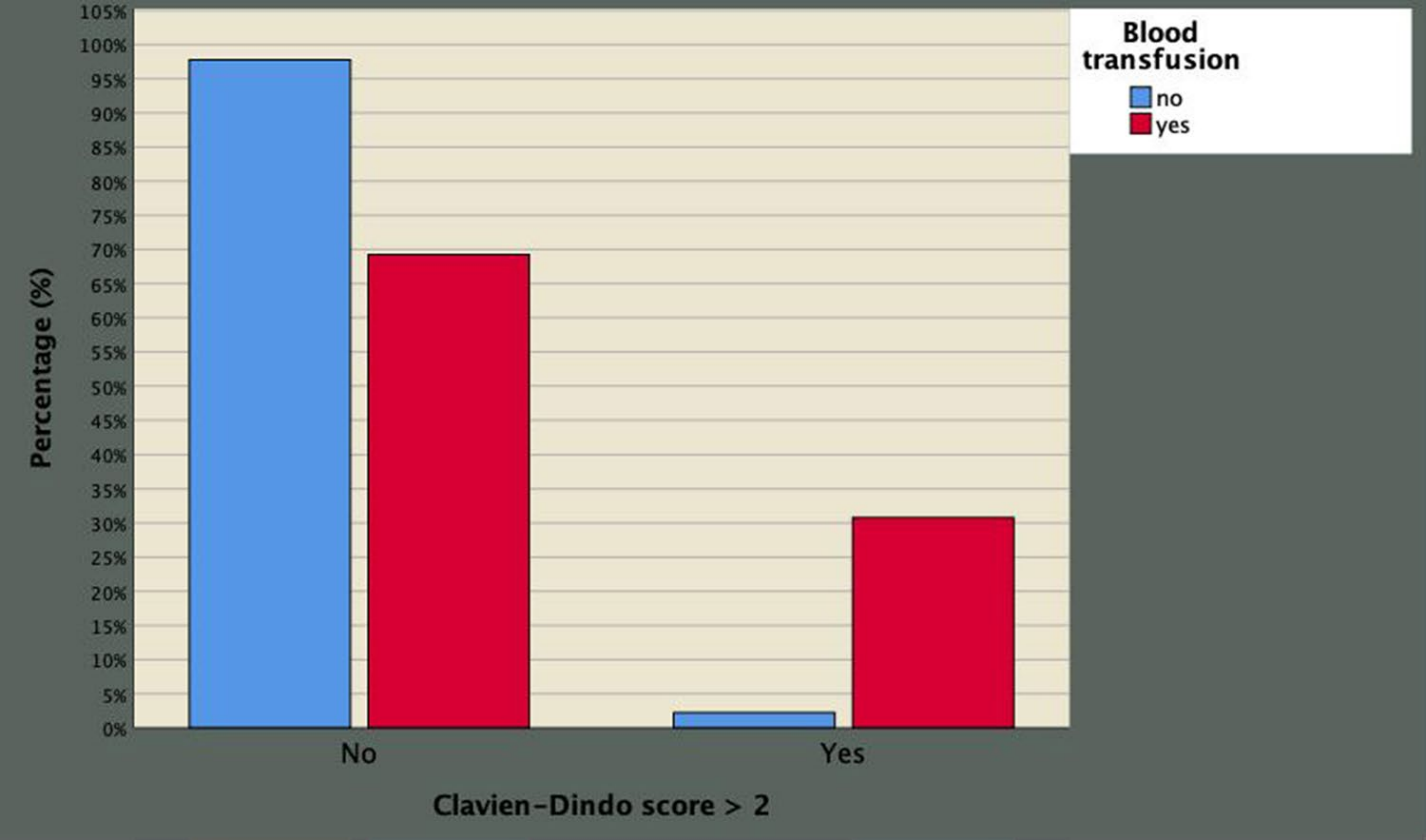
Blood transfusion is associated with major Clavien–Dindo complications in 980 consecutive patients who underwent robot-assisted radical prostatectomy as a primary treatment for prostate cancer (corrected odd ratio, OR 4.036 with 95% CI 1.239 to 13.148)

On multivariate analysis, perioperative blood transfusion was associated with both prolonged LOHS OR 1.633; 95% CI 1.411–1.889; *p* < 0.0001) as well as with Clavien–Dindo complications greater than two (OR 4.036; 95% CI 1.239–13.148; *p* = 0.021). The implications of perioperative blood transfusion on delayed LOHS as well on major Clavien–Dindo complications are shown in Figs. 5, 6, respectively. The majority of procedures were performed by high-volume surgeons and no significant differences in blood loss were found according to surgeon experience.

## Discussion

Guidelines on reporting and grading of complications after urological surgical procedures recommend the use of standardized systems such as the Clavien–Dindo system. Here, blood transfusions are classified as grade 2 complications [4, 15]. Additionally, the Accordion system classifies postoperative complications according to their severity. In both classifications, blood transfusions are coded as a moderate complication [4]. However, when a postoperative complication is classified as grade 2, it is not specific because it may represent an adverse event requiring medical treatment [4].

The association of blood transfusion with RARP surgery is a topic that has been investigated only as a report within the set of postoperative complications. A review of 12 studies, published between 2006 and 2009, reported on RARP perioperative outcomes showed that blood transfused rates ranged from 0.5 to 5.1% [17]. Furthermore, the review pointed out that RARP decreased the risk of transfusion when compared with open radical prostatectomy due to the lower intraoperative blood loss; however, the authors did not examine the factors predicting blood transfusion in RARP surgery [17]. In North America, several studies investigating the National Surgery Quality Improvement Program (NSQIP) were conducted. They include a large USA PCA population treated by open or minimally invasive radical prostatectomy (MIRP), which includes both laparoscopic and RARP procedures [18–21]. Pilecki et al. while comparing 30-day postoperative complication and readmission rates in 5471 NSQIP patients, reported blood transfusion rates of 1.9% in RARP cases [18]. Xia et al. studied pre-discharge predictors of readmissions and post-discharge complications in 9975 NSQIP patients who underwent RARP showing a blood transfusion rate of 1.2% [19]. Pereira et al. also investigated on perioperative morbidity and mortality in the NSQIP population that included 35,968 patients and found out a positive association between age, 30-day complications and perioperative morbidity [20]. Specifically, blood transfusion rates were significantly higher in men aged 70 to 89 years compared to men aged less than 60 years old (6.0% versus 3.7%). Notably, the population was not stratified by surgical approach since 29,024 cases were classified as MIRP, which included both laparoscopic and RARP cases [20]. Britto III et al. again investigated 29,012 NSQIP patients who underwent MIRP. Authors found that overall blood transfusion rates of 1.7%, without a difference between patients who did or did not undergo a lymph node dissection [21]. However, studies utilizing the NSQIP database suffer several limitations, indeed they are retrospective studies not including surgeon characteristics and pathology findings [18–21]. In another large study, *Leow and associates* investigated on a cohort of 629,593 men who underwent radical prostatectomy at 449 hospitals in the USA from 2003 to 2013. They detected significantly lower blood transfusion rates in RARP patients compared to open radical prostatectomy (0.3% versus 2.9%). One of the main limits of the study was the underestimated minor complication rates of grade 1–2 according to the Clavien–Dindo system [22]. A European controlled study including a contemporary cohort of 110 individuals undergoing RARP with or without ePLND showed that overall blood transfusion rates were low (1.8%) and was not associated with the decision of whether or not to place a drain in the pelvic cavity [23]. In a Swedish multicenter prospective comparative trial including 3706 patients, Walerstedt Lantz et al. evaluated postoperative complications and 90-day re-admission rates comparing open and RARP cases and found that the latter were significantly less likely to be transfused compared to the former (16% versus 4%) which included data from both high- and low-volume centers [24]. So far, perioperative blood transfusion rates range from 0.5% to 5.1%, which are in line with the results of our study showing a perioperative transfusion rate of 4% that included both intraoperative (*n* = 4; 0.4%), and postoperative transfused cases (*n* = 35; 3.6%), as well.

We investigated among potential factors predicting the risk of and found that only severe intraoperative bleeding was associated with the risk perioperative blood transfusion in a contemporaneous large tertiary center cohort. This is the first study showing evidence that the amount of intraoperative blood loss may impact the risk of perioperative blood transfusion, which is low but not negligible for the related implications.

The risk of perioperative blood transfusion is associated with severe intraoperative bleeding which results in a prolonged operating time and makes the surgery more challenging. Patients with elevated BMI have a particularly elevated risk. Patients who underwent perioperative blood transfusion were at increased risk of prolonged LOHS and to severe Clavien–Dindo complications, as well. The increased risk of bleeding in obese patients might be explained by the hypothesis that periprostatic fat tissue is present in greater quantity and is associated with an increased number of vessels. As such, the prostate dissection becomes more challenging resulting in a higher risk of severe intraoperative bleeding; furthermore, coagulative disorders might occur more frequently in obese patients who are at increased risk of more aggressive PCa and major Clavien–Dindo complications, as well [25, 26].

Severe intraoperative bleeding with perioperative blood transfusion, might carry potential oncological drawbacks. The hemorrhage may obscure the view of the tumor-resulting in a less accurate dissection of the tumor with a higher risk of positive surgical margins and an increased risk of cancer recurrence and progression due to immune system suppression [24, 27]. The results of our study suggest that patients should be counseled on the risk of perioperative blood transfusion and the implications related to this unfavorable event. Obese patients remain in a special category who may require a more challenging surgery while carrying the increased risk of perioperative blood transfusion, less accurate oncological surgery and increased risk of cancer recurrence and progression as well as lymph node invasion after open and or robotic procedures [28, 29]. The risk of blood transfusion is also associated with increased hospital costs for both prolonged LOHS and major Clavien–Dindo complications; as a result, a robotic prostate surgery in these patients may not carry all the advantages intended.

Our study has several limits. First, it was retrospective and, as such, suffers of the limitations of these kind of studies. Second, it was single center and as such, limited for the single cohort. Third, operations were not performed by a single surgeon but by two groups classified as low- and high-volume surgeons and this might be a bias. Additionally, according to our internal protocol, patients were discharged in 4th post-operative day if complications did not occur, and it could influence the association of clinical factors and length of hospital stay.

Although our study has limits, it also has strengths. First, although it was retrospective, data were prospectively collected. Second, although it was single center, the cohort was homogenous and belonged to a tertiary reference center, which is a referral center for urologic robotic surgery.

## Conclusion

In patients undergoing RARP, the risk of perioperative blood transfusion represented a rare event predicted by severe intraoperative bleeding, which was associated with increased BMI as well as with prolonged operating time.

## Supplementary Information

Below is the link to the electronic supplementary material.

Supplementary file1 (XLSX 10 KB)

Supplementary file2 (XLSX 10 KB)

Supplementary file3 (XLSX 10 KB)

Supplementary file4 (XLSX 11 KB)
